# A metanalysis to evaluate the effects of substrate sources on the nutritional performance of black soldier fly larvae: implications for sustainable poultry feed

**DOI:** 10.1016/j.psj.2023.103299

**Published:** 2023-11-18

**Authors:** Abdolreza Hosseindoust, Sang Hun Ha, Jun Young Mun, Jin Soo Kim

**Affiliations:** Department of Animal Industry Convergence, Kangwon National University, Chuncheon 24341, Korea

**Keywords:** bioconversion, survivability, larval, waste reduction, efficiency

## Abstract

This meta-analysis presents an evaluation of substrate sources and their impact on the growth performance of black soldier fly (**BSF**) larva. The database, compiled from Google Scholar, PubMed, and Science Direct, focuses on data concerning substrate sources, environmental conditions, and the performance parameters of BSF. Seven types of substrates were analyzed, including Feed Waste, Manure, Fruits, Mix, Animal Source, Fermentation Residue, and Food Waste. The Feed Waste group demonstrated the highest DM content, while the highest CP content was found in the Animal Source group. Higher CP and DM content were found in larva meal from Fermentation Residues and Feed Waste diets, respectively. Higher survival rates were observed in BSF larvae fed on Feed Waste, Fermentation Residues, Food Waste, Fruits, Mix, and Manure substrates compared to Vegetable and Animal Source substrates. Fresh larval weight was lower when Manure was used as a feed substrate than in the Animal Source, Feed Waste, and Vegetable substrates. The prepupal Wet Weight was highest in BSF larvae fed on Animal Source, surpassing those fed on Fermentation Residue, Manure, and Vegetable substrates. Substrate CP content exhibited a positive relationship with fresh larva weight, prepupal wet weight, dry larval weight; larval length, mortality until prepupal, protein conversion, feed conversion ratio, food consumption, substrate reduction rate bioconversion ratio, waste reduction index, and efficiency conversion of digested feed in BSF larva. In conclusion, our findings underline that the source and composition of substrates are correlated to the nutritional composition and conversion efficiency of BSF larva meal.

## INTRODUCTION

The black soldier fly (**BSF**), *Hermetia illucens*, is an insect species that has gained considerable attention in the past years due to its potential for organic waste recycling and sustainable animal feed production (Dzepe et al., 2021; Zhao et al., 2022). The larvae of this fly exhibit remarkable voracity for organic matter and have the ability to bioconvert waste materials into nutrient-rich biomass suitable for use as poultry feed (Meneguz et al., 2018; Kim et al., 2020a; Yang et al., 2022; Facey et al., 2023). The nutritional composition of BSF larvae is highly dependent on the substrate used for their rearing, a factor that is thus crucial for the quality and suitability of the resulting BSF larva meal for specific applications (Grossule and Lavagnolo, 2020; Hopkins et al., 2021; Luparelli et al., 2022). Although BSF larvae are known to have a broad dietary range, the precise effects of substrate composition on larval growth, nutrient composition, and bioconversion efficiency remain not completely understood. Previous research has largely been confined to individual substrate types (Meneguz et al., 2018; Fuso et al., 2021; Isibika et al., 2021; Hosseindoust et al., 2023b), and thus far, a comprehensive, comparative analysis across a wide range of substrates has been lacking.

In this study, we aimed to address this gap by conducting an analysis of the impacts of various substrate types on the performance and composition of BSF larva meal. We examined 7 categories of substrates including Feed Waste, Manure, Fruits, mix of substrates (MIXX), Animal Source, Fermentation Residue, and Food Waste, and evaluated their effects on the nutritional composition of BSF larvae. Moreover, we elucidated the conversion efficiency of these substrates into BSF larva meal, providing insights into their potential for sustainable waste management and poultry feed production. Additionally, we investigated the relationships between substrate and environmental parameters and the composition of BSF larva meal. We aimed to provide a deeper understanding of the factors influencing the nutrient profile of BSF larvae, ultimately contributing to the optimization of BSF rearing practices for waste conversion and poultry feed production purposes. By elucidating these dynamics, our findings provided a significant contribution to the growing body of knowledge surrounding BSF biology, and more importantly, provided practical insights for those seeking to harness the potential of these versatile insects in various applications.

## MATERIALS AND METHODS

### Database Description

A database was compiled from a corpus of 65 peer-reviewed papers published in scholarly journals between the years 2005 to 2022. These papers were sourced from prominent academic search engines and databases including Google Scholar, PubMed, and Science Direct. The database primarily focused on encompassing data related to substrate sources, environmental conditions, and the growth performance of BSF. For a study to be incorporated into the final database, it was required to meet a set of stringent criteria. These criteria included: 1) clear identification of substrate sources, 2) explicit reporting of the final BSF meal composition, 3) measurable BSF performance parameters, and 4) experiments conducted under rigorously controlled temperature and humidity conditions. It is pertinent to note that all these criteria were carefully selected to ensure the highest level of data quality and relevance. However, it was observed that the reported values for all variables were not uniformly available across all observations. As a result, the count of observations employed for statistical analyses varied among response variables. In certain cases, records were found to be incomplete or inconsistently reported, necessitating additional calculations based on the available data. When a study failed to report the outcome for all variables or when it was infeasible to deduce a value from the presented data, these missing values were treated under the assumption of missing at random. This strategy was undertaken to mitigate potential biases in statistical analysis and interpretation due to missing data, thereby preserving the robustness and reliability of our conclusions. The summary of the gathered data is shown in Table 1.

**Table 1:** Simple statistics of the measured parameters.

Variable	N	Mean	SD	Minimum	Maximum
Temperature	420	27.3	2.25	20	35
Humidity	351	66.8	5.2	50	77.6
Substrates					
DM	300	36.6	27.23	6.2	95.1
CP	322	21.2	13.95	3.4	86.1
CH	247	43.3	21.92	0.3	85.6
GE	157	3903	956.2	960	5570
EE	304	7.43	7.4	0.6	49.7
Fiber	211	17.5	11.86	0.7	58.4
ADF	68	27.1	17	5.9	57.7
NDF	72	40.9	17.72	7.8	75.1
BSF characteristics					
CP	265	41.5	6.99	21.2	57.6
DM	167	46.9	25	21	95.5
GE	13	4674	1032	3723	6650
EE	214	24.9	11.02	3.1	57.8
Fiber	7	6.34	2.58	4	10.3
ADF	16	12.5	6.39	6.48	27.4
NDF	12	21.2	9.75	8.7	39.1
Ash	121	10.5	6.79	2.7	33
LA	32	43.8	18.53	21.4	78.9
Chitin	16	7.83	6.48	1.4	23
SR	262	80.9	18.7	3.2	100
FLW	269	0.118	0.06	0	0.3
PWW	69	0.12	0.08	0.01	0.3
LL	39	16.3	2.88	11.14	22.4
MUL	21	16.4	7.54	4.58	29.6
MUP	14	25.5	13.8	8	54
PC	23	31.7	21.7	1.9	80.4
FCR	70	15	26.9	1.4	147
FC	59	6.96	11.1	0.6	43.2
SRR	140	55.1	15.8	12.7	84.8
BR	163	13.3	9.29	1.2	50.3
GR	27	0.01	0.01	0.001	0.04
WRI	69	1.8	1.43	0.4	5.3
ECDF	88	20	12.8	1.4	50

Abbreviations: ADF, acid detergent fiber; BCR, bioconversion ratio; BSF, black soldier fly; CH, carbohydrate; CP, crude protein; DLW, dry larval weight; DM, dry matter; ECDF, efficiency conversion of digested feed; EE, ether extract; FC, food consumption; FCR, feed conversion ratio; FLW, fresh larval weight; GE, gross energy; GR, growth rate; LL, larval length; MUL, mortality until larva; MUP, mortality until prepupal; NDF, neutral detergent fiber; PC, protein conversion; PWW, prepupal wet weight; SR, survival rate; SRR, substrate reduction rate; WRI, waste reduction index.

### Data Extraction and Quality Assessments

In this meta-analysis, the focus was on evaluating substrates for the growth of BSF during its larval and prepupal stages. We considered environmental parameters such as temperature and humidity that influence BSF growth. Substrates were categorized into 7 sections: Animal Sources, Fermentation Products, Food Waste, Fruits, Manure, Vegetables, and MIXX. The “Animal Source” category encompasses substrates including poultry slaughterhouse waste, fish, fresh mussels, ensiled mussels, rotten mussels, fish waste and soybean cake, fish offal diet, cricket waste, locust waste, swine liver, butchery waste, and fish rendering. “Feed Waste” substrates comprise wheat bran, soybean meal, barley, corn, sorghum, poultry feed, dried distillers grains with solubles, roasted soybean seeds, cornmeal, grain middling, digestate, grape pulp, potato pulp, bean seeds, sunflower oil, cabbage leaves, old bread, alfalfa, cow casein, sucrose, dog food, hen diet, chicken mash substrate, brewery waste, spent grain, Gainesville diet, chicken start mash, and beer draff. Fermentation Products include winery by-product, brewery by-product, maize distillers, brewers grains, brewery waste, brewer waste, tofu dreg, and grain from brewery waste. The “Food Waste” category incorporates substrates such as market waste, hotel waste, canteen waste, bread, restaurant waste, brown algae at various percentages, abattoir waste, banana peels, food remains, kitchen waste, industrial food waste, household food waste, okara, municipal organic waste, soybean curd residue, kitchen waste at different ratios, beef, pork, standard diet, white rice, curry rice, coconut milk rice, fried rice, and restaurant waste. The “Fruits” category covers apple, grape pomace, pumpkin, almond byproducts, a mixture of vegetable and fruit wastes (including pears, bananas, tomatoes, and various leafy green vegetables), fruit waste, fruit and vegetables, abattoir fruit and vegetable waste, apple pulp, fruit puree, banana peel, orange peel, and a combination of apple, banana, grain, and various fruits and vegetables. The “Manure” category consists of substrates such as human feces, cow manure, swine manure, dairy manure, poultry manure, primary sludge, undigested sludge, digested sludge, fecal sludge, chicken manure, dairy manure, quail manure, house manure, cattle manure, and various combinations of dairy manure at various percentages. The “Vegetables” category includes red cabbage, red onion, spinach, vegetable canteen waste, vegetable waste, fermented maize straw, forced chicory roots, tomato leaves, vegetable overproduction auction, vegetable and fruits, vegetable by-product, fruits, vegetable diet, semi-digested grass, and total vegetable diet. The “Mixed Substrates” or MIXX group refers to those which contained substrates from different categories, with none of the individual substrates accounting for more than 50% of the total composition. Additionally, the analysis took into a BSF food composition characteristics, which include moisture, DM, CP, carbohydrates, gross energy (**GE**), ether extract (**EE**), fiber, acid detergent fiber (**ADF**), neutral detergent fiber (**NDF**), ash, and pH. We also considered the composition of the BSF itself, including its CP, DM, GE, EE, crude fiber, ADF, NDF, ash, lauric acid, and chitin content. Lastly, we evaluated the performance of the BSF across several metrics: larval weight (**LW**; g/larva), larval length (**LL**), prepupae length (**PL**), mortality until larval stage (**MUL**), mortality until prepupal stage (**MUP**), wet conversion (**WC**), DM conversion, protein conversion (**PC**), nitrogen conversion (**NC**), prepupal wet weight (**PWW**), feed conversion ratio (**FCR**), food consumption (**FC**), substrate reduction rate (**SRR**), bioconversion ratio (**BR**), growth rate (**GR**), waste reduction index (**WRI**), and efficiency of conversion of digested feed (**ECDF**).

### Statistical Analyses

In this research, the statistical analysis of the collected data was carried out using the SAS software (SAS Inst. Inc., Cary, NC) to evaluate the potential random effects introduced by the study and their potential interactions with fixed effect factors. The MIXED procedure was employed as an advanced method of analysis. This procedure allowed us to consider the interaction between 2 crucial growth stages—the larval stage and the prepupal stage. Alongside this, we examined 7 distinct substrate groups: Animal Sources, Fermentation Products, Food Waste, Fruits, Manure, Vegetables, and Mixed Substrates. For each substrate composition subgroup, a correlation analysis was conducted to discern the relationships between substrate types and the BSF composition or performance. This examination allowed for a meticulous exploration of the various correlations between different substrate components and their impacts on BSF characteristics. In addition, further statistical methods, such as subgroup and regression analyses, were deployed to assess the influence of substrate composition and survival rate on the final outcomes.

## RESULTS

### Composition of Substrates

The comprehensive details of these substrate compositions are presented in Table 2. Notably, the Feed Waste group exhibited the highest content of DM, surpassing that found in Manure, Fruits, and MIXX groups. The CP content, on the other hand, was observed in the Animal Source group, which also surpassed the levels in the Feed Waste, Fruits, and MIXX groups. The Carbohydrate content varied among the groups, being highest in Fruits and lowest in the Animal Source, Fermentation Residue, Food Waste, Mix, and Manure groups. The Food Waste and Manure groups showed higher Carbohydrate content compared to Fermentation Residue and MIXX groups. As for Gross Energy, it was higher in the Animal Source group than in the Feed Waste, MIXX, Manure, and Vegetable groups. Similarly, Fermentation Residue, Food Waste, and Fruits showed elevated gross energy levels compared to Manure and Vegetables. Animal Source led in terms of EE content, surpassing Food Waste, MIXX, Feed Waste, Fruits, Manure, and Vegetables. Furthermore, the MIXX group showed the highest fiber content, followed by Manure and Vegetables, and then the Animal Source, Feed Waste, Food Waste, and Fruits groups. The ADF content was higher in the Fruits group compared to Animal Source, Feed Waste, Food Waste, and Vegetables. Contrastingly, the ADG was noticeably higher in Fermentation Residue than in the Feed Waste group. NDF content was higher in the MIXX group compared to the Animal Source group. Ash content was found to be highest in the Manure group, also surpassing the levels in the Animal Source, Feed Waste, Fermentation Residues, Food Waste, Fruits, and MIXX groups. The pH level was found to be highest in the Manure group, exceeding those in the Animal Source, Feed Waste, Fruits, and Vegetables groups.

**Table 2:** The composition of BSF feed substrates.

Group	DM	CP	CH	GE	EE	Fiber	ADF	NDF	Ash	pH
Animal source	43.6 ± 5.8^b,c^	40.3 ± 2.5a	21.5 ± 4.1^c^	4998 ± 464^a^	19.8 ± 1.4^a^	3.9 ± 2.8^d^	13.4 ± 8.3^b,c^	23.6 ± 9.5^b^	7.7 ± 2.0^c^	5.1 ± 0.7^b^
Feed waste	64.0 ± 4.3^a^	24.2 ± 1.8^b^	49.1 ± 3.4^a,b^	3908 ± 205^b,c,d^	4.9 ± 0.9^d^	14.8 ± 2.0^c^	15.5 ± 5.8^c^	38.6 ± 6.6^a,b^	8.0 ± 0.8^c^	5.2 ± 0.5^b^
Fermentation residues	48.7 ± 9.0^b,c^	18.1 ± 4.5^b,c^	18.4 ± 6.9^c^	4737 ± 395^a,b^	7.5 ± 2.2^c,d^	-	32.4 ± 7.8^a,b^	50.8 ± 8.0^a,b^	8.1 ± 1.8^c^	-
Food waste	42.4 ± 4.8^b,c^	24.4 ± 2.7^b^	35.8 ± 4.4^b^	4417 ± 371^a,b,c^	13.2 ± 1.4^b^	13.2 ± 2.8^c^	16.5 ± 6.5^b,c^	28.5 ± 7.2^a,b^	7.1 ± 1.4^c^	6.0 ± 0.3^a,b^
Fruits	34.1 ± 3.9^c^	13.3 ± 1.8^c^	59.4 ± 3.0^a^	4209 ± 224^a,b,c^	5.8 ± 0.9^d^	14.6 ± 1.8^c^	38.7 ± 5.0^a^	47.0 ± 5.7^a,b^	8.1 ± 1.0^c^	5.6 ± 0.7^b^
MIXX	37.8 ± 8.1^c^	15.6 ± 4.0^c^	18.3 ± 6.9^c^	3508 ± 491^c,d^	10.8 ± 1.9^b,c^	44.1 ± 3.6^a^	22.9 ± 14.9^a,b,c^	52.3 ± 17.0^a^	9.4 ± 1.6^c^	6.2 ± 1.0^a,b^
Manure	54.9 ± 4.5^b^	20.8 ± 2.4^b,c^	35.5 ± 4.5^b^	2966 ± 267^d^	4.5 ± 1.3^d^	26.3 ± 3.3^b^	27.5 ± 7.8^a,b,c^	45.5 ± 8.9^a,b^	19.2 ± 1.3^a^	7.7 ± 0.2^a^
Vegetable	46.3 ± 4.8^b,c^	16.1 ± 2.2^b,c^	50.0 ± 4.0^a,b^	3169 ± 367^d^	5.1 ± 1.2^d^	18.1 ± 2.2^b,c^	25.1 ± 7.4^b,c^	32.2 ± 8.4^a,b^	14.7 ± 1.4^b^	4.4 ± 0.6^b^
*P*-value	<0.001	<0.001	<0.001	<0.001	<0.001	<0.001	<0.001	<0.001	<0.001	<0.001

^a-d^ Means in the same coulmn with different superscript differ significantly (P < 0.05).

Abbreviations: ADF, acid detergent fiber; CH, carbohydrate; CP, crude protein; DM, dry matter; EE, ether extract; GE, gross energy; MIXX, a mix of substrates; NDF, neutral detergent fiber.

### Composition of Black Soldier Fly Larva Meal

Detailed compositions of BSF larva meal are provided in Table 3. In terms of CP content, BSF larva meal, resulting from the Fermentation Residues diet, showed higher levels compared to the Food Waste, Mix, and Manure diets. The Feed Waste diet also demonstrated a high CP content, surpassing MIXX and Manure diets. The DM content was higher in larva meal from the Feed Waste diet, compared to those fed on Animal Source, Fermentation Residue, Food Waste, Mix, and Vegetable diets. Additionally, the Fruits diet exhibited a higher DM content relative to the Fermentation Residue diet. The GE content was considerably higher in BSF larva meal produced from the Fruits diet as opposed to the Food Waste diet. The EE content was notably higher in the Food Waste diet compared to Feed Waste, Fruits, MIXX, Manure, and Vegetables diets. Furthermore, higher EE content was observed in the Animal Source and Fermentation Residue diets than in the MIXX diet. For ADF content, a higher level was detected in BSF larva meal resulting from the Fruits diet compared to the Animal Source diet. Interestingly, there was no change in NDF content across all treatment groups. Ash content was markedly elevated in BSF larva meals from the MIXX and Vegetable diets, outstripping those from the Feed Waste, Fermentation Residue, Food Waste, Fruits, and Vegetable diets. Furthermore, the lauric acid content in BSF meal was highest when the larvae were fed on Feed Waste. In terms of chitin content, a higher level was noted in the Fruits diet compared to Food Waste and Feed Waste diets.

**Table 3:** Effects of the source of substrates on the composition of BSF larva meal.

	CP	DM	GE	EE	ADF	NDF	Ash	LA	Chitin
Animal source	41.9 ± 1.8^a,b,c^	43.1 ± 6.8^b,c^	5551 ± 741^a,b^	29.6 ± 2.9^a,b^	4.1 ± 4.2^b^	11.2 ± 6.9	12.3 ± 1.9^a,b^	59.3 ± 7.8^b^	–
Feed waste	44.4 ± 0.9^a,b^	62.8 ± 4.6^a^	4627 ± 929^a,b^	23.1 ± 1.5^b,c^	17.2 ± 5.6^a,b^	32.0 ± 9.1	8.1 ± 1.0^b^	56.5 ± 2.8^a^	1.7 ± 4.1^b^
Fermentation residues	46.2 ± 2.4^a^	44.7 ± 9.1^c^	–	31.1 ± 3.7^a,b^	12.4 ± 3.4^a,b^	16.6 ± 6.9	8.2 ± 2.4^b^	–	7.5 ± 3.2^a,b^
Food waste	39.9 ± 1.2^b,c^	50.1 ± 5.3^b,c^	3200 ± 667^b^	34.4 ± 2.4^a^	12.6 ± 3.6^a,b^	17.2 ± 5.9	8.2 ± 1.4^b^	46.6 ± 3.3^b^	2.5 ± 5.3^b^
Fruits	42.2 ± 1.4^a,b,c^	58.9 ± 8.6^a,b^	5844 ± 485^a^	22.7 ± 2.3^b,c^	18.5 ± 2.6^a^	29.1 ± 4.5	9.5 ± 1.6^b^	45.0 ± 5.2^b^	14.4 ± 2.5^a^
MIXX	37.9 ± 1.8^c^	46.5 ± 8.5^b,c^	–	21.3 ± 3.9^c^	–	–	15.9 ± 1.8^a^	–	11.0 ± 4.1^a,b^
Manure	38.4 ± 1.2^c^	56.1 ± 5.8^a,b,c^	5057 ± 626^a,b^	22.1 ± 2.4^b,c^	16.8 ± 5.6^a,b^	25.3 ± 9.1	16.7 ± 2.8^a^	54.1 ± 4.0^b^	–
Vegetable	42.2 ± 1.6^a,b,c^	42.8 ± 7.8^b,c^	–	23.6 ± 3.0^b,c^	15.5 ± 5.6^a,b^	–	10.4 ± 2.0^b^	52.2 ± 5.8^b^	6.0 ± 3.4^a,b^
*P*-value	<0.001	<0.001	<0.001	<0.001	<0.001	0.106	<0.001	<0.001	<0.001

^a-c^ Means in the same coulmn with different superscript differ significantly (P < 0.05).

Abbreviations: ADF, acid detergent fiber; CH, carbohydrate; CP, crude protein; DM, dry matter; EE, ether extract; GE, gross energy; MIXX, a mix of substrates; NDF, neutral detergent fiber.

### The Performance and Conversion Efficiency of Black Soldier Fly Larva Meal

The effect of various substrate sources on the performance and conversion efficiency of BSF larva meal is depicted in Tables 4 and 5. Higher survival rates were observed in BSF larvae fed on Feed Waste, Fermentation Residues, Food Waste, Fruits, MIXX, and Manure substrates compared to Vegetable and Animal Source substrates. Fresh LW was lower when Manure was used as a feed substrate than in the cases of Animal Source, Feed Waste, and Vegetable substrates. The prepupal wet weight was highest in BSF larvae fed on Animal Source, surpassing those fed on Fermentation Residue, Manure, and Vegetable substrates. Furthermore, higher prepupal wet weight was noted in larvae fed on Feed Waste, Food Waste, and Fruits compared to Vegetables. The prepupal dry weight was highest when the larvae were fed Manure, while the lowest weight was recorded for larvae fed Vegetables. The LW was higher in Fermentation Residues-fed larvae than those fed on Animal Source, Fruits, MIXX, Manure, and Vegetable substrates. Moreover, LL was higher in larvae fed Fermentation Residues than those fed Animal Source, Fruits, and Manure substrates. Interestingly, larvae fed Animal Source had a greater length than those fed Manure. The prepupal length was higher in larvae fed on Feed Waste and Food Waste than those fed on Fruits. No change was observed in DM conversion across the treatments. However, protein conversion was higher in larvae fed on Feed Waste than those fed Manure and Fermentation Residues. The SRR was notably higher in larvae fed Fruits compared to those fed Feed Waste, Manure, and Vegetable substrates. Also, the SRR was higher in the MIXX group compared to the Vegetable group. The highest BR was noted for larvae fed on Fermentation Residues. The BR was higher in larvae fed Animal Sources, Feed Waste, Food Waste, MIXX, and Manure compared to those fed Fruits. The WRI was higher in larvae fed on Fermentation Residues and Food Waste compared to those fed on Feed Waste, Fruits, and Vegetables. Moreover, the WRI was higher in larvae fed on Animal Source and Vegetables than those fed Fruits. The efficiency conversion of digested feed was highest in larvae fed on Animal Source compared to those fed Fruits.

**Table 4:** Effects of the source of substrates on the physical performance of BSF larva meal.

Group	SR	FLW	PWW	PDW	LW	LL	PL
Animal source	67.5 ± 5.3^b^	0.21 ± 0.01^a^	0.20 ± 0.05^a^	–	0.10 ± 0.03^b^	16.6 ± 1.1^b^	–
Feed waste	93.5 ± 5.0^a^	0.20 ± 0.05^a^	0.15 ± 0.02^a,b,c^	0.11 ± 0.01^b^	0.12 ± 0.01^a,b^	14.4 ± 0.9^b,c^	2.17 ± 0.01^a^
Fermentation residues	88.4 ± 9.3^a^	–	0.13 ± 0.03^b,c,d^	0.12 ± 0.01^b^	0.17 ± 0.03^a^	19.9 ± 2.0^a^	–
Food waste	88.3 ± 6.0^a^	–	0.15 ± 0.02^a,b,c^	0.13 ± 0.01^b^	0.12 ± 0.01^a,b^	17.4 ± 0.9^a,b^	1.96 ± 0.01^a^
Fruits	84.3 ± 4.6^a^	0.14 ± 0.01^a,b^	0.20 ± 0.04^a,b^	–	0.08 ± 0.01^b^	13.7 ± 2.0^b,c^	1.52 ± 0.01^b^
MIXX	90.4 ± 6.4^a^	–	–	–	0.10 ± 0.02^b^	–	–
Manure	88.1 ± 4.9^a^	0.10 ± 0.02^b^	0.09 ± 0.02^c,d^	0.09 ± 0.00^a^	0.07 ± 0.03^b^	13.5 ± 1.2^c^	–
Vegetable	66.3 ± 5.3^b^	0.20 ± 0.04^a^	0.05 ± 0.03^d^	0.08 ± 0.01^c^	0.10 ± 0.01^b^	16.1 ± 1.5^a,b^	–
*P*-value	<0.001	<0.001	<0.001	<0.001	<0.001	<0.001	<0.001

^a-d^ Means in the same coulmn with different superscript differ significantly (P < 0.05).

Abbreviations: DLW, dry larval weight; FLW, fresh larval weight; LL, larval length; LW, larval weight; MIXX, a mix of substrates; PL, prepupae length; PWW, prepupal wet weight; SR, survival rate.

**Table 5:** Effects of the source of substrates on the performance and conversion efficiency of BSF larva meal.

Group	DMC	PC	SRR	BCR	WRI	EFDG
Animal source	–	23.0 ± 17.7^a,b^	55.1 ± 4.0^a,b,c^	15.7 ± 2.8^b^	2.8 ± 0.7^a,b^	30.8 ± 6.9^a^
Feed waste	13.1 ± 15.0	50.9 ± 10.2^a^	49.3 ± 4.9^b,c^	16.0 ± 2.7^b^	1.7 ± 0.6^b,c^	22.6 ± 4.7^a,b^
Fermentation residues	–	10.3 ± 17.7^b^	51.7 ± 7.0^a,b,c^	31.3 ± 3.7^a^	3.9 ± 0.7^a^	23.3 ± 5.4^a,b^
Food waste	14.6 ± 15.0	35.4 ± 10.2^a,b^	56.3 ± 4.7^a,b,c^	17.5 ± 2.9^b^	3.9 ± 0.8^a^	20.0 ± 8.3^a,b^
Fruits	9.2 ± 15.0	41.0 ± 12.5^a,b^	64.3 ± 3.4^a^	8.4 ± 2.2^c^	1.1 ± 0.6^c^	9.4 ± 4.5^b^
MIXX	–	37.6 ± 12.5^a,b^	61.1 ± 5.6^a,b^	16.1 ± 2.8^b^	–	–
Manure	32.4 ± 5.7	16.1 ± 7.2^b^	48.1 ± 4.0^b,c^	14.8 ± 2.4^b^	–	17.7 ± 6.6^a,b^
Vegetable	–	36.0 ± 12.5^a,b^	47.3 ± 5.0^c^	13.9 ± 2.5^b,c^	1.4 ± 0.6^b,c^	17.7 ± 4.7^a,b^
*P*-value	0.166	0.016	<0.001	<0.001	<0.001	<0.001

^a-c^ Means in the same coulmn with different superscript differ significantly (P < 0.05).

Abbreviations: BCR, Bioconversion ratio; DMC, dry matter conversion; EFDG, Efficiency of conversion of digested feed; FCR, feed conversion ratio; MIXX, a mix of substrates; PC, protein conversion; SRR, Substrate reduction rate; WRI, Waste reduction index.

### Correlation Between Environment or Substrate Composition and Black Soldier Fly Larva Meal Composition

**Figure 1:**
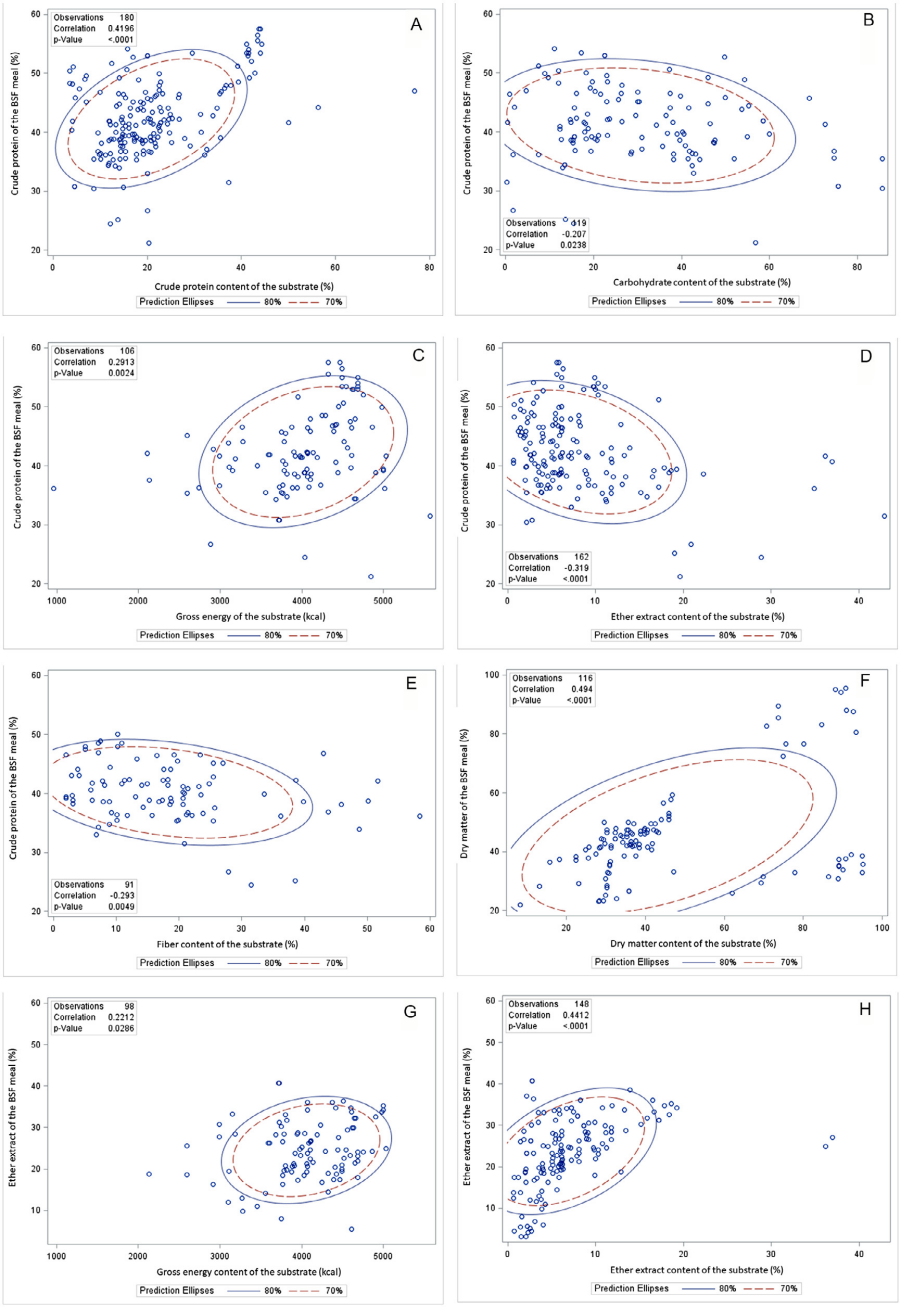

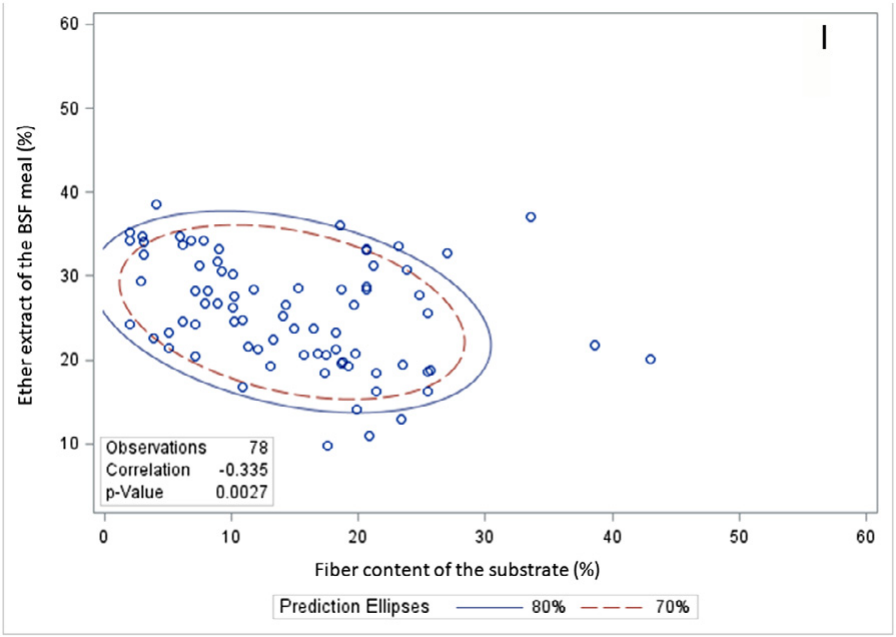
Pearson correlation coefficients, *P*-value, and the number of observations between growth environment or substrates composition and the composition of BSF larva meal. Abbreviation: BSF, black soldier fly.

In the examination of substrate composition and BSF larva meal composition, several correlations were discerned (Table 6, Figure 1). Elevated rearing temperature was found to positively influence the GE, ADF, NDF, and chitin constituents of the BSF larva meal. Concurrently, an increase in rearing humidity aligned with an enhancement in the GE, NDF, lauric acid, and chitin levels within the BSF larva meal. Significant associations were detected between substrate DM content and the DM, ADF, lauric acid, and chitin content in the BSF larva meal, underscoring a positive relationship. Similarly, a positive correlation was observed whereby higher CP content in the substrate coincided with increased levels of CP, GE, lauric acid, and chitin in the BSF larva meal. A rise in substrate carbohydrate content also showed a positive correlation with fiber, ADF, NDF, and chitin levels in the BSF larva meal. Furthermore, substrate gross energy content exhibited a positive correlation with the CP, GE, and EE in the BSF larva meal. EE content in the substrate showed a positive tendency to boost the GE, EE, ADF, lauric acid, and chitin content in the BSF larva meal. A positive correlation was noted between the fiber content of the substrate and the GE, ADF, ash, and chitin content in the BSF larva meal. A positive correlation was observed between the ADF content in the substrate and the DM, fiber, ADF, NDF, ash, and chitin content in the BSF larva meal. The analysis concluded with the identification of a positive link between substrate NDF content and the CP, DM, ADF, NDF, ash, lauric acid, and chitin content in the BSF larva meal.

**Table 6:** Pearson correlation coefficients, *P*-value, and the number of observations between growth environment or substrates composition and the BSF larva meal composition.

		Environmental condition and substrates composition									
		Temperature	Humidity	DM	CP	CH	GE	EE	Fiber	ADF	NDF
BSF larva meal composition	CP	−0.100	−0.034	0.121	0.420	−0.207	0.291	−0.319	−0.293	0.101	0.255
		0.129	0.617	0.139	<0.001	0.024	0.002	<0.001	0.005	0.571	0.123
		233	216	150	180	119	106	162	91	34	38
	DM	−0.014	0.053	0.494	−0.249	0.020	−0.273	−0.059	0.060	0.390	0.358
		0.865	0.529	<0.001	0.005	0.865	0.009	0.530	0.619	0.136	0.121
		143	143	116	124	79	90	116	72	16	20
	GE	−0.038	−0.038	0.846	0.587	−0.872	0.705	0.748	−0.090	–	–
		0.929	0.929	0.154	0.126	0.005	0.051	0.033	0.910	–	–
		8	8	4	8	8	8	8	4	0	0
	EE	0.007	−0.043	−0.046	0.043	0.064	0.221	0.441	−0.335	−0.286	−0.239
		0.930	0.566	0.630	0.605	0.538	0.029	<0.001	0.003	0.147	0.196
		184	178	113	150	95	98	148	78	27	31
	Fiber			0.827	−0.458	0.946	–	−0.164	0.790	–	–
				0.173	0.542	0.054	–	0.836	0.210	–	–
		0	0	4	4	4	0	4	4	0	0
	ADF	0.242	0.159	0.429	−0.344	0.334	−0.622	−0.375	–	0.441	0.320
		0.366	0.558	0.097	0.192	0.315	0.100	0.206	–	0.087	0.228
		16	16	16	16	11	8	13	0	16	16
	NDF	0.425	0.358	0.325	−0.517	0.462	−0.585	−0.419	–	0.586	0.449
		0.169	0.253	0.302	0.086	0.297	0.415	0.262	–	0.045	0.143
		12	12	12	12	7	4	9	0	12	12
	Ash	−0.152	−0.296	−0.169	−0.344	−0.173	−0.385	−0.338	0.345	0.465	0.398
		0.153	0.005	0.240	0.004	0.319	0.027	0.006	0.126	0.006	0.013
		90	90	50	69	35	33	66	21	34	38
	LA	−0.635	0.202	0.549	−0.322	–	–	0.229	–	–	–
		<0.001	0.294	0.015	0.334	–	–	0.498	–	–	–
		29	29	19	11	0	0	11	0	2	2
	Chitin	–	0.134	0.844	−0.345	0.530	−0.576	−0.317	−0.991	0.382	0.226
		–	0.622	<0.001	0.190	0.177	0.081	0.231	0.009	0.275	0.530
		16	16	16	16	8	10	16	4	10	10

Abbreviations: ADF, acid detergent fiber; CH, carbohydrate; CP, crude protein; DM, dry matter; EE, ether extract; GE, gross energy; LA, lauric acid; NDF, neutral detergent fiber.

### Correlation Between Environmental and Substrate Composition and Black Soldier Fly Larva Performance

**Figure 2:**
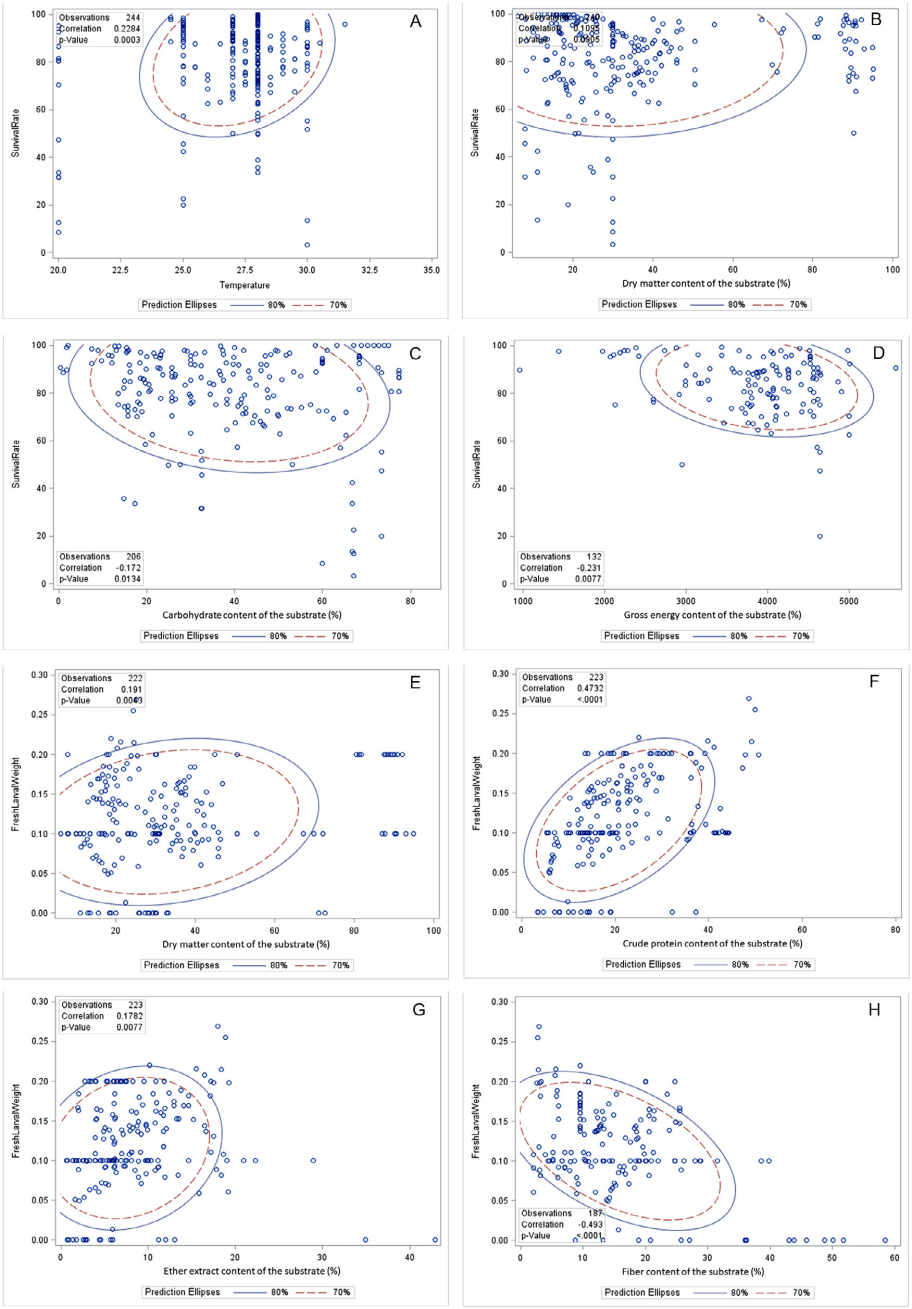
Pearson correlation coefficients, *P*-value, and the number of observations between growth environment or substrates composition and growth performance of BSF larva. Abbreviation: BSF, black soldier fly.

**Figure 3:**
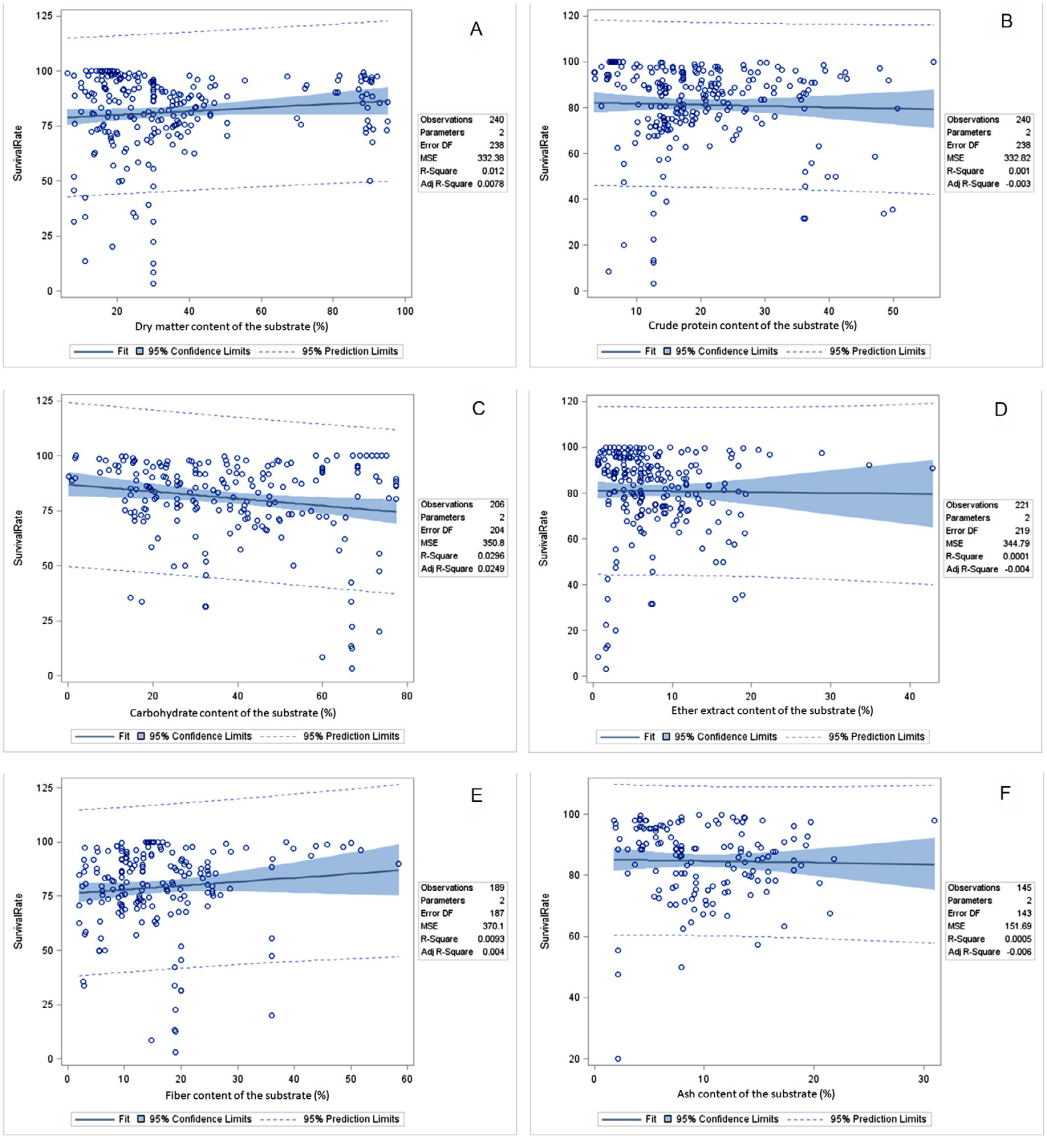
The regression coefficients, R-square, mean standard error, and the number of observations between growth environment or substrates composition and survival rate of BSF. Abbreviation: BSF, black soldier fly.

**Figure 4:**
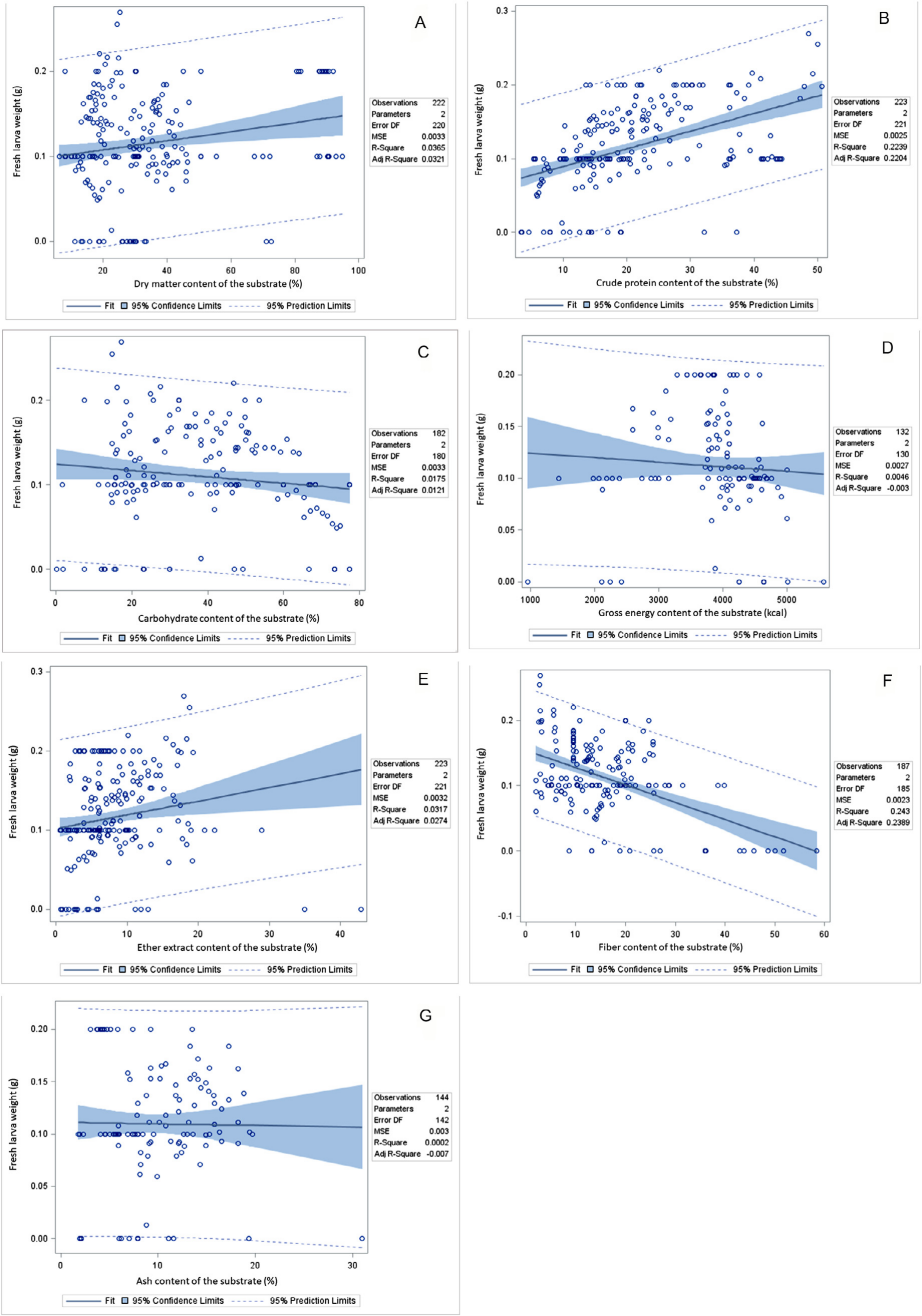
The regression coefficients, R-square, mean standard error, and the number of observations between growth environment or substrates composition and fresh larva weight of BSF. Abbreviation: BSF, black soldier fly.

The correlation between environmental and substrate compositions and the performance of BSF larvae can be seen in Table 7 and Figures 2, 3, and 4. A positive correlation emerged between rearing temperature and various aspects of BSF larva performance, including SR, FLW, DLW, LL, MUL, MUP, PC, FC, SRR, BCR, GR, WRI, and ECDF. Similarly, rearing humidity positively influenced FLW, PC, FCR, FC, SRR, BCR, GR, WRI, and ECDF of the BSF larva. The DM content in substrates was positively linked with FLW, DLW, MUL, PC, FC, BCR, WRI, and ECDF in the BSF larva meal. However, a negative correlation was identified between substrate DM content and the SRR. Substrate CP content exhibited a positive relationship with various parameters such as FLW, PWW, DLW, LL, MUP, PC, FCR, FC, SRR, BCR, WRI, and ECDF in BSF larva. Substrate carbohydrate content showed positive correlations with PWW, LL, MUP, PC, and SRR in BSF larva meal. However, it demonstrated a negative association with FLW and BCR in the BSF larva. Substrate GE content was positively correlated with PWW, LL, FCR, SRR, and GR in BSF larva. Substrate EE content exhibited a positive correlation with a broad range of performance indicators, such as FLW, PWW, LL, MUP, PC, FCR, FC, BCR, WRI, and ECDF in BSF larva. Fiber content in substrates was associated positively with LL, MUP, FCR, FC, BCR, and GR in BSF larva meal but negatively with DLW in BSF larva. Substrate ADF content was found to positively affect FLW, LL, MUL, MUP, PC, and FCR in BSF larva meal. Substrate NDF content had positive correlations with FLW, LL, MUL, FCR, and GR in BSF larva meal, but it was negatively correlated with WRI in BSF larva.

**Table 7:** Pearson correlation coefficients, *P*-value, and the number of observations between growth environment or substrates composition and growth performance of BSF larva.

	Temperature	Humidity	DM	CP	CH	GE	EE	Fiber	ADF	NDF
SR	0.228	−0.031	0.110	−0.032	−0.172	−0.231	−0.012	0.097	−0.488	−0.310
	<0.001	0.675	0.091	0.627	0.013	<0.001	0.862	0.186	0.001	0.027
	244	184	240	240	206	132	221	189	47	51
FLW	0.117	0.071	0.191	0.473	−0.132	−0.068	0.178	−0.493	−0.306	−0.309
	0.065	0.315	0.004	<0.001	0.075	0.438	0.008	<0.001	0.062	0.047
	251	202	222	223	182	132	223	187	38	42
PWW	−0.310	−0.305	−0.275	0.143	0.175	−0.163	0.002	0.908	−0.778	−0.578
	0.021	0.044	0.141	0.453	0.503	0.612	0.995	0.092	0.433	0.607
	55	44	30	30	17	12	16	4	3	3
DLW	0.267	−0.163	−0.250	0.592	−0.415	0.752	0.660	−0.970	−0.835	−0.655
	0.116	0.343	0.589	0.008	0.355	0.001	0.005	0.030	0.371	0.545
	36	36	7	19	7	16	16	4	3	3
LL	–	−0.465	0.526	−0.374	−0.095	−0.171	−0.026	−0.475	0.600	0.318
	–	0.052	0.053	0.188	0.769	0.559	0.930	0.526	0.116	0.443
	18	18	14	14	12	14	14	4	8	8
MUL	0.513	−0.558	0.432	−0.476	0.291	−0.431	−0.428	−0.397	0.060	−0.303
	0.107	0.075	0.286	0.233	0.484	0.287	0.290	0.603	0.940	0.698
	11	11	8	8	8	8	8	4	4	4
MUP	0.463	0.861	0.489	0.013	0.582	−0.682	0.390	–	−0.997	−0.877
	0.040	0.003	0.055	0.961	0.047	0.205	0.517	–	0.053	0.319
	20	9	16	16	12	5	5	0	3	3
PC	−0.299	0.140	−0.247	0.544	−0.284	0.585	0.343	0.419	0.734	0.706
	0.015	0.262	0.053	<0.001	0.048	<0.001	0.014	0.003	<0.001	<0.001
	66	66	62	56	49	33	51	49	29	29
FCR	0.234	–	0.484	−0.206	0.199	−0.404	−0.298	−0.162	−0.074	−0.057
	0.110	–	0.001	0.170	0.174	0.050	0.040	0.305	0.769	0.822
	48	42	42	46	48	24	48	42	18	18
FC	0.099	0.238	−0.386	0.063	0.389	0.313	−0.066	−0.284	−0.189	−0.236
	0.266	0.049	<0.001	0.511	<0.001	0.092	0.535	0.014	0.293	0.186
	129	69	116	110	100	30	91	75	33	33
SRR	0.249	0.440	0.147	0.324	−0.547	−0.118	0.505	0.090	−0.500	−0.492
	0.002	<0.001	0.098	<0.001	<0.001	0.445	<0.001	0.347	0.001	0.002
	151	102	127	121	116	44	121	110	38	38
BCR	0.308	0.356	0.154	−0.108	−0.064	0.516	0.081	–	0.197	0.187
	0.144	0.147	0.598	0.671	0.802	0.191	0.733	–	0.586	0.521
	24	18	14	18	18	8	20	0	10	14
GR	0.196	0.197	0.388	0.092	−0.302	−0.423	0.292	−0.527	−0.513	−0.661
	0.115	0.113	0.003	0.495	0.022	0.016	0.032	<0.001	0.004	<0.001
	66	66	57	57	57	32	54	42	29	33
WRI	0.252	0.235	0.432	0.173	−0.150	−0.085	0.005	−0.522	−0.357	−0.367
	0.020	0.037	0.002	0.212	0.279	0.673	0.971	0.002	0.073	0.046
	85	79	50	54	54	27	53	33	26	30
ECDF	0.228	−0.031	0.110	−0.032	−0.172	−0.231	−0.012	0.097	−0.488	−0.310
	<0.001	0.675	0.091	0.627	0.013	0.008	0.862	0.186	0.001	0.027
	244	184	240	240	206	132	221	189	47	51

Abbreviations: ADF, acid detergent fiber; BCR, bioconversion ratio; CH, carbohydrate; CP, crude protein; DLW, dry larval weight; DM, dry matter; ECDF, efficiency conversion of digested feed; EE, ether extract; FC, food consumption; FCR, feed conversion ratio; FLW, fresh larval weight; GE, gross energy; GR, growth rate; LL, larval length; MUL, mortality until larva; MUP, mortality until prepupal; NDF, neutral detergent fiber; PC, protein conversion; PWW, prepupal wet weight; SR, survival rate; SRR, substrate reduction rate; WRI, waste reduction index.

## DISCUSSION

### Composition of Substrates

The variation in DM, CP, Carbohydrates, GE, EE, Fiber, ADF, NDF, ash, and pH across different substrate groups validates the flexibility of the BSF larval diet requirement, and its direct impact on their biochemical composition. Feed Waste displayed the highest DM content, which may be attributable to the inherent properties of the feed itself, often characterized by high organic and nutrient content (English et al., 2021). The Animal Source group exhibited the highest CP and EE content, which is expected given that animal-based substrates typically have high protein levels. The high GE content in the Animal Source group and the comparably elevated levels in Fermentation Residue, Food Waste, and Fruits suggest that these substrates provide substantial energy to the larvae. This observation indicates that larvae fed with these substrates can potentially serve as high-energy feed ingredients, and increase the meal value as a protein source for poultry. Ash content was highest in the Manure group, reflecting the mineral-rich composition of manure (Spranghers et al., 2017; Ewald et al., 2020).

### Composition of Black Soldier Fly Larva Meal

High CP content was found in BSF larvae meals resulting from the Fermentation Residues, Animal source, and Feed Waste diets, which suggests that these substrates could contain higher quality proteins or nitrogenous compounds than Manure (Raksasat et al., 2020) to be used in poultry diets. This observation aligns with the existing understanding that the CP content of BSF larvae meal can be manipulated based on the protein quality of the substrate provided (Chia et al., 2021; Seyedalmoosavi et al., 2022). Fermentation Residues, Animal Source, and Feed Waste substrates likely possess a more complex and diverse set of proteins or nitrogenous compounds than Manure. The diverse range of proteins can ensure a balanced amino acid profile, improving the overall protein quality of the larvae meal (Meneguz et al., 2018). Moreover, the potentially higher bioavailability of proteins in these substrates can lead to more efficient protein assimilation by the BSF larvae, further boosting the CP content of the resulting meal to be used efficiently in poultry diet. This superior efficiency can then translate to a higher CP content in the larvae meal, marking the direct influence of substrate quality on meal composition (Villa et al., 2021; Eggink et al., 2023). These observations emphasize the potential to manipulate the CP content of BSF larvae meal by selecting substrates with higher protein quality. Utilizing high-quality protein substrates can lead to larvae meal with enhanced nutritional profiles, thereby augmenting its value as a sustainable protein source in poultry feed.

The higher DM content found in BSF larvae reared on Feed Waste and Fruits diets underscores the significance of substrate organic matter quality in shaping larvae meal composition. Organic matter quality in a substrate refers to the presence and accessibility of bioavailable nutrients, energy-dense compounds, and beneficial microorganisms (Deng et al., 2022). Feed Waste and Fruits substrates typically contain a diverse array of digestible organic matter, including carbohydrates, proteins, fats, and micronutrients (Meneguz et al., 2018; Fuso et al., 2021; Hopkins et al., 2021). The BSF larvae, utilizing their efficient bioconversion capabilities, assimilate these nutrients effectively, resulting in high DM content in the final meal (Rehman et al., 2017; Lopes et al., 2020). BSF larvae exhibit an exceptional capacity for nutrient assimilation, transforming organic matter in their substrate into body mass, which depends on the quality of the substrate (Meneguz et al., 2018; Villa et al., 2021). High-quality substrates like Feed Waste and Fruits, rich in digestible organic matter, can enhance the larvae nutrient assimilation efficiency. This superior assimilation translates into a high DM content in the larvae meal, demonstrating the direct influence of substrate quality on the nutrient density of the meal (Rehman et al., 2017; Lopes et al., 2020). Employing substrates rich in diverse, bioavailable nutrients can yield larvae meal with enhanced DM content, thereby increasing its value as a nutrient-dense feed ingredient.

The higher GE content in the Fruits diet-derived BSF larva meal emphasizes the crucial role of substrate nutrient density on the energy composition of larvae meal. Gross energy content represents a vital indicator of the nutritive value of BSF larvae meal, as it reflects the total calorific value or energy available. Fruits are renowned for their high sugar content, which represents a primary source of easily digestible, energy-rich nutrients. When these fruits are used as a substrate, BSF larvae can efficiently assimilate these high-energy compounds, translating to an elevated GE content in the resulting larvae meal. The bioconversion efficiency of BSF larvae, or their capability to convert substrate nutrients into body mass, impacts the GE content (Seyedalmoosavi et al., 2022). As fruits are replete with simple sugars and other energy-rich nutrients, they can enhance larvae bioconversion efficiency, which subsequently elevates the GE content in larvae meal (Fuso et al., 2021). This finding aligns with existing research affirming the prowess of BSF larvae to convert a variety of substrates into energy-dense biomass. The efficient utilization of high-energy substrates like fruits testifies to the versatility and adaptability of BSF larvae in diverse dietary conditions (Dzepe et al., 2021; Scieuzo et al., 2023). These observations underline the potential for manipulating the GE content of BSF larvae meal by selecting substrates with high nutrient density, such as fruits. Using energy-rich substrates can lead to larvae meal with an enhanced GE content, thereby augmenting its value as an energy source in poultry feed.

The EE content, indicative of the total fat in the larva meal, was higher in the Food Waste, Animal Source, and Fermentation Residue diets. This result suggests these substrates could contain a high amount of fats or lipids, leading to a high-fat BSF larva meal (Giannetto et al., 2020; Seyedalmoosavi et al., 2022). The fruits diet led to a higher ADF content in BSF larva meal compared to the Animal Source diet. This finding could be linked to the higher cellulose and lignin content present in fruits, which is transformed into ADF in the BSF larva body (Isibika et al., 2019). Interestingly, there was no variation in the NDF content across all the treatment groups. This result suggests that the BSF larvae have a similar capacity to process different substrates into NDF, which comprises the cell wall components of plants, including cellulose, hemicellulose, and lignin. Elevated ash content was observed in the BSF larva meals from the MIXX and Vegetable diets. Ash content in the insect meal generally reflects the mineral content of the diet, suggesting that the MIXX and vegetable substrates were potentially rich in minerals.

The BSF larva meal resulting from the Feed Waste diet showed the highest lauric acid content known for its antimicrobial properties (Ewald et al., 2020; Kim et al., 2020b). This could be due to specific components present in the feed waste that enhance the synthesis of lauric acid in BSF larvae. Lastly, the fruits diet resulted in a higher chitin content compared to the Food Waste and Feed Waste diets. This finding could be related to the specific nutritional composition of fruits that possibly stimulates chitin production in BSF larvae (Luparelli et al., 2022). These results highlight the importance of substrate selection in BSF rearing, which directly impacts the nutritional profile of the resulting larvae meal. It offers insights for tailoring BSF larva meal as a sustainable feed ingredient for poultry.

### The Performance and Conversion Efficiency of Black Soldier Fly Larva

The choice of substrate in BSF larva rearing presents profound implications for the performance and conversion efficiency of the resulting larva meal (Villa et al., 2021; Seyedalmoosavi et al., 2022; Eggink et al., 2023). The present meta-analysis reveals substantial variation in larva survival rates, larval and prepupal weights, and lengths, and efficiency indices associated with different substrate sources. The elevated survival rates witnessed in larvae fed on Feed Waste, Fermentation Residues, Food Waste, Fruits, MIXX, and Manure substrates compared to Vegetable and Animal Source substrates indicate the relative nutritional suitability and palatability of these substrates for BSF larvae. These substrates possibly present a more balanced composition of macro and micronutrients, as well as a diverse array of bioavailable compounds that can cater to the broad nutritional requirements of the growing larvae (Lopes et al., 2022). BSF larvae are recognized for their omnivorous feeding habits, thus, a diversified substrate (Singh et al., 2021; Villa et al., 2021; Luparelli et al., 2022), offering a broad spectrum of nutrients, may be beneficial for their growth and survival. It also signifies the role of substrate quality and nutrient profile in influencing the survival rate (Rehman et al., 2017; Meneguz et al., 2018; Lopes et al., 2020), demonstrating the need for careful substrate selection based on the intended outcomes of the BSF rearing process. Interestingly, survival rate differences observed across substrates hint towards the adaptability of BSF larvae to a variety of substrates, but also reflect their selective feeding preference based on the nutritional content, which might affect their physiological resilience. The higher survival rates on certain substrates might also result from a healthier gut microbiome (Kar et al., 2021; Foysal and Gupta, 2022; Seyedalmoosavi et al., 2022), induced by the nutrients in the substrates, which plays a vital role in the health and survival of insects . However, it is essential to understand that survival rate, though a key parameter in insect rearing, is not a holistic indicator of the overall success or efficiency of the rearing process. It should be evaluated in conjunction with other performance metrics such as growth rate, prepupal weight, bioconversion efficiency, and SRR to obtain a comprehensive view of the BSF-rearing process.

The prepupal wet weight was highest in BSF larvae fed on Animal Source substrates, suggesting efficient utilization of animal-derived nutrients for growth and development. Interestingly, while manure led to the highest prepupal dry weight, it contributed to lower fresh LW compared to other substrates like Animal Source, Feed Waste, and Vegetable substrates, indicating potential variations in the moisture content of the substrates affecting the weight metrics (Bekker et al., 2021; Hosseindoust et al., 2023a). The LL was higher in larvae fed Fermentation Residues than those fed on other substrates, implying the potential of Fermentation Residues to promote larval elongation. Similarly, higher prepupal lengths were noted in larvae fed on Feed Waste and Food Waste substrates, further affirming the correlation between substrate type and larval growth metrics (Meneguz et al., 2018; Grossule and Lavagnolo, 2020; Luparelli et al., 2022). The SRR was higher in larvae fed Fruits and MIXX substrates. In line with this, the highest BR was observed in Fermentation Residues-fed larvae, indicating efficient substrate conversion to biomass in this group. Finally, the WRI was markedly higher in larvae fed on Fermentation Residues and Food Waste, signifying their prowess in waste reduction. Also, the efficiency conversion of digested feed was highest in larvae fed on Animal Source, implying superior digestibility and nutrient assimilation from these substrates.

### Correlation Between Environment or Substrate Composition and Black Soldier Fly Larva Meal Composition

Elevated rearing temperatures positively correlated with the GE, ADF, NDF, and chitin content of the BSF larva meal. This suggests a potential enhancement in energy extraction and chitin production processes within the larvae at higher temperatures. Similarly, increased rearing humidity demonstrated a positive correlation with the GE, NDF, lauric acid, and chitin content, indicating a potential impact of moisture levels on nutrient absorption and metabolism within the larvae (Bekker et al., 2021).

A strong correlation was detected between the CP content in the substrate and the protein, GE, lauric acid, and chitin content within the BSF larva meal. In the context of protein transformation, the BSF exhibit a considerable capability to assimilate and bio-convert proteins present in the substrate into their own body mass (Meneguz et al., 2018; Dzepe et al., 2021; Eggink et al., 2023; Hosseindoust et al., 2023a). This inherent biological function, in part, explains the observed correlation between substrate protein content and the protein levels in the resulting larva meal. The observed correlation between substrate protein content and the GE in larva meal could be attributable to the inherent energy content of proteins (Meneguz et al., 2018; Eggink et al., 2023). It is well-established that proteins contribute to the energy value of feedstuffs, with an energy yield comparable to that of carbohydrates and considerably lower than lipids (Villa et al., 2021; Seyedalmoosavi et al., 2022). Therefore, a substrate with a higher protein content is likely to yield larva meal with elevated GE content (Meneguz et al., 2018; Eggink et al., 2023), as reflected in our findings. Furthermore, the correlation between substrate protein content and the lauric acid content in larva meal is noteworthy. Lauric acid plays crucial roles in energy provision and other metabolic functions (Meneguz et al., 2018). Although the precise mechanism driving this relationship warrants further investigation, it could be speculated that a high-protein diet might affect the lipid metabolism of BSF larvae, leading to an increase in certain fatty acids like lauric acid. Finally, the observed relationship between substrate protein content and chitin levels in BSF larva meal is intriguing. The link between protein and chitin content may be related to the larvae metabolic processes, whereby higher protein input could facilitate or stimulate chitin synthesis (Pener and Dhadialla, 2012). The Gross Energy content in the substrate exhibited a strong relationship with the CP, GE, and EE content in the BSF larva meal, signifying that energy-dense substrates may lead to the production of larva meal with higher caloric content. Furthermore, the EE content in the substrate showed a positive tendency to augment the GE, EE, ADF, lauric acid, and chitin content in the BSF larva meal. This aligns with the understanding that fat-rich substrates could boost the energy content (Giannetto et al., 2020; Kim et al., 2020a; Seyedalmoosavi et al., 2022) and potentially enhance the lipid and chitin composition of the BSF larva meal.

### Correlation Between Environmental and Substrate Composition and Black Soldier Fly Larva Performance

The positive correlation between rearing temperature and a wide array of performance parameters suggests that BSF larvae thrive under higher temperatures, which could be attributable to the tropical origin of these insects (Kim et al., 2020b). Elevated temperature possibly optimizes their metabolic activities, consequently enhancing survival rates, growth characteristics (fresh LW, dry LW, and LL), and their capability to convert the substrate into biomass (protein conversion and BR). Rearing humidity showed a similar positive correlation with various performance indicators, indicating its crucial role in BSF larvae overall well-being and productivity. High humidity levels prevent excessive moisture loss from the larvae (Bekker et al., 2021), thereby promoting growth and feed conversion efficiency. This could be explained by the fact that substrates with high DM content might be more difficult for the larvae to break down and metabolize, reducing the overall SRR (Meneguz et al., 2018; Matin et al., 2021).

The strong positive correlation observed between substrate CP content and multiple performance parameters substantiates the crucial role of protein-rich substrates in promoting BSF larval growth and bioconversion efficiency (Meneguz et al., 2018; Fuso et al., 2021; Hopkins et al., 2021). This pattern indicates that larval development and the transformation of substrates into valuable biomass is protein-dependent, with protein-rich feeds stimulating better growth and bioconversion responses (Meneguz et al., 2018; Giannetto et al., 2020; Fuso et al., 2021; Hosseindoust et al., 2023a). This outcome corroborates previous studies that underline the capability of BSF larvae to utilize proteinaceous wastes (Giannetto et al., 2020; El-Dakar et al., 2021; Eggink et al., 2023), thereby converting these materials into a high-quality protein source in their biomass. This process, in effect, emphasizes the crucial role of the substrate nitrogenous compounds in the life cycle of the BSF, particularly in its growth phase. The BSF larvae unique biological machinery allows them to biochemically break down and incorporate the nitrogen from protein-rich substrates into their own body tissues (Meneguz et al., 2018; Villa et al., 2021; Eggink et al., 2023). In a broader context, the enrichment of larval feed with proteins could lead to the elevation of essential amino acids in the produced BSF larva meal, enhancing its nutritional profile (Giannetto et al., 2020; El-Dakar et al., 2021). This could potentially translate into an enhanced quality of the insect meal, which could be beneficial in application for poultry feed where the protein quality is of paramount importance. However, it is noteworthy that the efficiency of protein conversion may also depend on the bioavailability and balance of amino acids present in the substrate, hinting towards the complexity of protein metabolism in BSF larvae. Consequently, a deeper understanding of the BSF larva protein requirements, substrate amino acid profile, and their digestibility is needed to optimize protein bioconversion. The positive influence of protein-rich substrates on the performance of BSF larvae accentuates the potential of BSF as a sustainable solution for managing proteinaceous wastes, while simultaneously producing a protein-rich biomass that can serve as a potential feedstock for various applications. Nevertheless, further research is needed to optimize the protein utilization efficiency of BSF larvae and to elucidate the exact mechanisms underlying their protein metabolism.

The substrate carbohydrate content and EE content also displayed positive correlations with certain performance indicators, suggesting their contributions to the nutritional quality of the substrate. However, the negative correlation observed between carbohydrate content and fresh LW and BR indicates that an overabundance of carbohydrates could potentially deter optimal larval growth and bioconversion performance (Meneguz et al., 2018; Eggink et al., 2023). Fiber content in the substrate, including ADF and NDF, showed mixed impacts on larval performance. While they positively affected several performance parameters, they negatively influenced dry LW and WRI. The discrepancy could be due to the fact that while fiber provides substrate structure and aids in larval movement, excessive fiber limit nutrient availability, hindering optimal larval growth and waste reduction (Meneguz et al., 2018). The complex interplay between rearing conditions and substrate composition underscores the importance of an approach when optimizing BSF larvae-rearing practices.

## CONCLUSIONS

In summary, this meta-analysis provides insights into the significant influence of substrate choice on the performance and conversion efficiency of BSF larva meal. Substrate attributes, like CP and carbohydrates, exhibited affirmative connections with fresh larval weight, prepupal wet weight, larval length, protein conversion, substrate reduction rate, and waste reduction index. By harnessing these findings, future BSF-rearing practices can be optimized to achieve desired outcomes in terms of the mix of substrates in proper ratios. Moreover, there were correlations between the rearing temperature and humidity, substrate composition, and the nutritional composition of BSF larva meal, offering insights for optimizing BSF larva-rearing strategies. These findings underscore the practical implications of substrate choice in refining BSF cultivation for diverse applications based on substrate-larval dynamics and emphasize the need for tailored substrate strategies to be suitable for poultry feed.
